# Integrated evaluation of umbilical cord mesenchymal stromal cell-derived extracellular vesicles across skin-related cellular and zebrafish models

**DOI:** 10.3389/fbioe.2026.1939569

**Published:** 2026-09-24

**Authors:** Peijun Zhao, Dan Xiao, Jingyu Xu, Qian Li, Yue Fang, Shiyun Xu, Feidi Xiao, Yaqi Qin, Yifu Wei, Yunfeng Gao, Cheguo Cai

**Affiliations:** 1 Henan Engineering Technology Research Center of Food Processing and Circulation Safety Control, College of Food Science and Technology, Henan Agricultural University, Zhengzhou, China; 2 Shenzhen Beike Biotechnology Co., Ltd., Shenzhen, China; 3 Shenzhen Beikeyuan Cell Technology Co., Ltd., Shenzhen, China; 4 Key Laboratory of Systems Health Science of Zhejiang Province, School of Life Science, Hangzhou Institute for Advanced Study, University of Chinese Academy of Science, Hangzhou, China

**Keywords:** biological activity, oxidative defense, Sebum control, skin health, UC-MSC-EVs

## Abstract

**Introduction:**

Mesenchymal stromal cell-derived extracellular vesicles (MSC-EVs) have shown biological effects in skin-related models, particularly in oxidative injury, wound repair, and photoaging. However, previous studies have used different MSC sources, EVs preparations, and experimental systems, and the activity of a defined EVs preparation across multiple skin-related processes has rarely been examined within a single study. Effects on sebocyte lipid metabolism and androgen-related responses are particularly poorly characterized.

**Methods:**

Human umbilical cord mesenchymal stromal cell-derived extracellular vesicles (UC-MSC-EVs) were characterized and evaluated across complementary cellular and zebrafish models assessing oxidative injury, keratinocyte migration and barrier-related proteins, UVB-induced fibroblast senescence and extracellular matrix responses, sebocyte lipid and androgen metabolism, melanogenesis, and dermal papilla cell function.

**Results:**

UC-MSC-EVs reduced UVB- and tert-butyl hydroperoxide-induced injury in HaCaT keratinocytes, decreased oxidative stress, and restored antioxidant enzyme activities. They also promoted keratinocyte migration, increased filaggrin and aquaporin-3 expression, and enhanced zebrafish tail-fin regeneration, increasing relative regenerated length from 60.24 ± 4.01% in the model group to 84.10 ± 5.82% at 48 h post-amputation. In UVB-exposed human skin fibroblasts, UC-MSC-EVs reduced cellular senescence and modulated extracellular matrix, inflammatory, and antioxidant responses. In oleic acid-stimulated SZ95 sebocytes, UC-MSC-EVs reduced lipid accumulation and lipid peroxidation and attenuated 5α-reductase activity and dihydrotestosterone production. In B16F10 cells stimulated with α-MSH, melanin content decreased from 147.60 ± 15.98% to 99.70 ± 7.98% of the untreated control at the highest tested UC-MSC-EVs concentration, accompanied by reduced tyrosinase activity and melanogenic gene expression. Pigmentation intensity was also reduced in zebrafish. In human dermal papilla cells, UC-MSC-EVs enhanced migration and increased alkaline phosphatase activity to 148.22 ± 4.72% of the control level, while increasing expression of hair growth factors and reducing androgen metabolism.

**Discussion:**

These results characterize the biological activity of a single UC-MSC-EVs preparation across multiple skin-related experimental systems and provide a basis for further investigation of its bioactive components and dose-response relationships.

## Graphical Abstract

**Figure:**
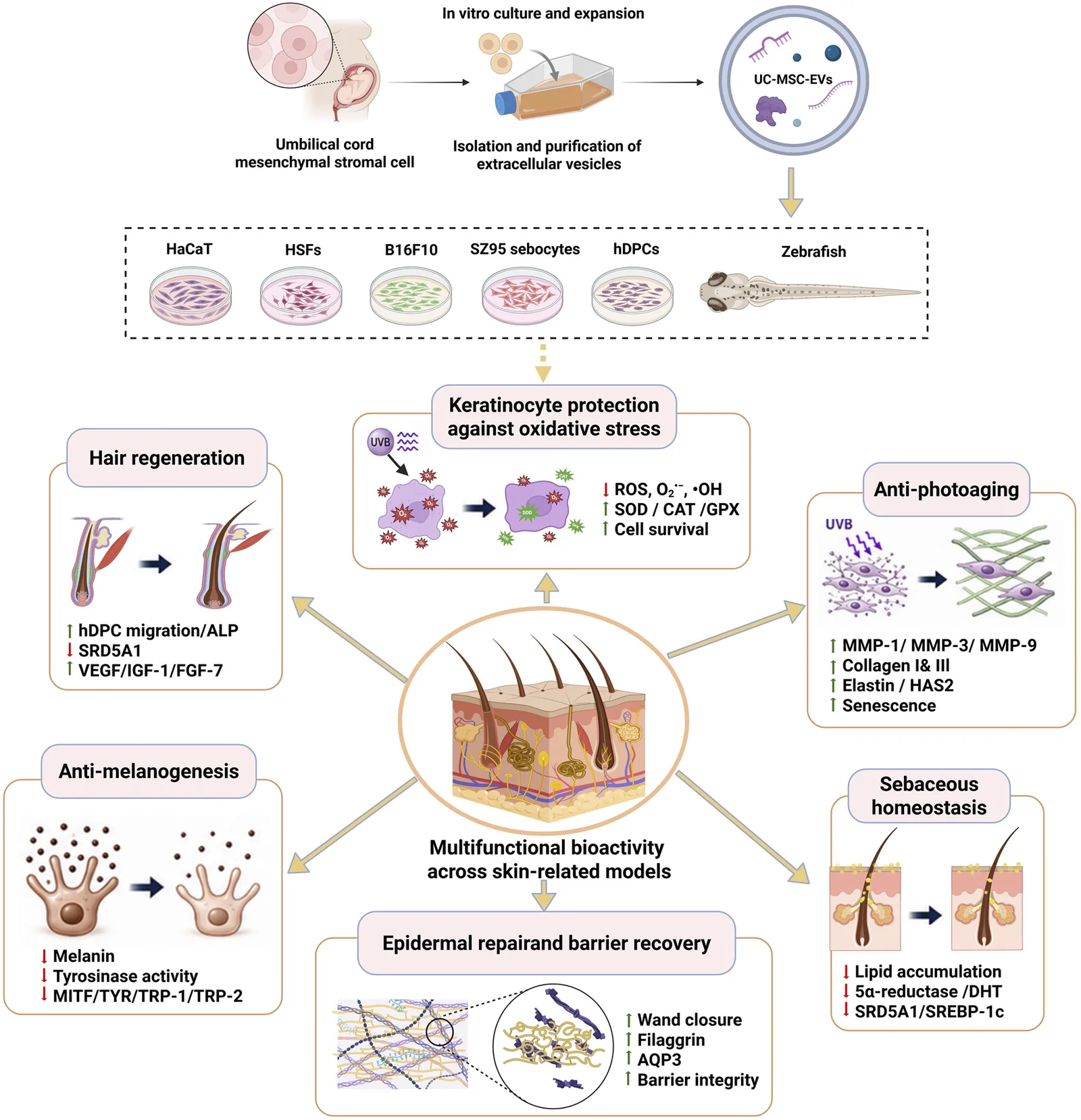

## 1 Introduction

The skin is a highly specialized organ that serves as the body’s primary barrier against environmental insults and helps maintain hydration and tissue integrity (Proksch et al., 2008). Cutaneous pigmentation contributes to photoprotection and local skin homeostasis (Slominski et al., 2004). Environmental exposures, particularly ultraviolet radiation and oxidative stress, can disrupt epidermal barrier function, extracellular matrix (ECM) organization, sebaceous activity, melanogenesis, and hair follicle-associated cellular functions (Krutmann et al., 2017; Kammeyer and Luiten, 2015). These changes contribute to common skin-related conditions and phenotypes, including photoaging, sebaceous dysfunction, hyperpigmentation, and hair loss (Ritti et al., 2015; Salminen et al., 2022). Although these conditions have distinct pathophysiological features, some of the underlying stress and regulatory processes may affect multiple components of the skin. Thus, skin dysfunction often involves changes in multiple biological processes rather than a single isolated alteration.

Current approaches to skin damage and dysfunction are generally designed for specific indications or biological targets. Mesenchymal stromal cell-derived extracellular vesicles (MSC-EVs) are membrane-bound particles carrying proteins, lipids, and nucleic acids that can modify recipient-cell functions (Welsh et al., 2024). As a cell-free product of MSCs, EVs may offer practical advantages for storage, handling, and formulation compared with viable-cell administration (Phinney and Pittenger, 2017). Human umbilical cord-derived MSCs are an attractive source for EVs production because the tissue can be obtained non-invasively and the cells exhibit high proliferative capacity (Nagamura-Inoue and He, 2014). These features have prompted increasing investigation of human umbilical cord MSC-EVs (UC-MSC-EVs) in regenerative and skin-related models.

Previous studies have demonstrated biological effects of MSC-derived EVs in several skin-related experimental settings. Liu et al. reported that UC-MSC-derived EVs enhanced HaCaT cell repair and reduced ultraviolet B (UVB)-induced cellular injury (Liu et al., 2021). Hu et al. showed that adipose-derived MSC EVs accelerated cutaneous wound healing by promoting fibroblast migration, proliferation, and collagen synthesis (Hu et al., 2016). Gao et al. further demonstrated that miR-1246-enriched adipose-derived MSC EVs alleviated UVB-induced photoaging through GSK3β-related autophagy regulation (Gao et al., 2024). More recent studies have extended UC-MSC-EVs research to ECM regulation and pigmentation (Kee et al., 2025). However, the available evidence has been generated using different MSC sources, EV preparations, experimental models, treatment conditions, and outcome measures, making it difficult to define the broader biological activity profile of a given UC-MSC-EVs preparation within a single study framework. In addition, sebaceous gland homeostasis is strongly influenced by lipid metabolism and androgenic regulation (Clayton et al., 2019; Del Rosso and Kircik, 2024). However, compared with wound repair and anti-aging, effects of MSC-EVs on sebocyte lipogenesis, lipid peroxidation, and androgen metabolism remain less extensively investigated.

Against this background, the present study evaluated human umbilical cord mesenchymal stromal cell-derived extracellular vesicles (UC-MSC-EVs) across a panel of complementary skin-related cellular and zebrafish models. Specifically, we investigated oxidative stress in keratinocytes, epidermal repair and barrier proteins, zebrafish tail-fin regeneration, UVB-induced fibroblast senescence and ECM remodeling, sebocyte lipogenesis and androgen metabolism, melanogenesis, and dermal papilla cell function. The objective was to determine the range of biological responses produced by the same UC-MSC-EVs preparation across these different experimental systems, with particular attention to the less studied effects on sebocyte lipid metabolism and androgen-related responses. By integrating these complementary models within a single study, we sought to obtain a broader view of the skin-related biological activities of UC-MSC-EVs.

## 2 Materials and methods

### 2.1 UC-MSC culture, EVs isolation, and characterization

UC-MSCs and UC-MSC-EVs were provided by the Shenzhen Beike Biotechnology Co., Ltd. (Shenzhen, China). All cellular materials used in this study were derived from a single donor, and only passage 4 (P4) UC-MSC were employed for cell cultivation and extracellular vesicles (EVs) preparation throughout the experiments.

UC-MSC were cultured in complete medium (Biological Industries) supplemented with 1% human platelet lysate, which was incubated at 37 °C in a humidified atmosphere of 5% CO_2_. When the cells reached approximately 80% confluency, adherent UC-MSC and culture supernatants were separately collected for cell characterization and EVs preparation.

For EVs preparation, the culture supernatant was sequentially centrifuged at 300 *g* for 10 min and 2,000 × g for 10 min to remove dead cells and debris. The supernatant was further centrifuged at 10,000 × g for 30 min and then ultracentrifuged at 100,000 × g for 90 min using an ultracentrifuge (Beckman Coulter). The pellet was resuspended in precooled phosphate-buffered saline (PBS) and subjected to a second ultracentrifugation at 100,000 × g for 90 min. The final pellet was resuspended in PBS and filtered through a 0.22 μm sterile filter to obtain purified UC-MSC-EVs. The morphology of UC-MSC-EVs were examined with a Hitachi H-7650 transmission electron microscope (Tokyo, Japan). Particle concentration and size distribution were analyzed using nanoparticle tracking analysis (NTA) on a NanoSight NS300 instrument (Malvern Instruments Ltd., Malvern, United Kingdom). The protein content of the UC-MSC-EVs was quantified with a Pierce BCA protein assay kit (Thermo Scientific, United States). EVs-associated proteins CD9, CD63, TSG101, ALIX, and the non-EVs cellular proteins GM130 and Calnexin were assessed by Western blotting.

The phenotypes of the UC-MSC were analysed via flow cytometry after incubation with CD90-BV421, CD29-PE, CD105-APC, CD73-PE, CD45-FITC, CD34- BV421, CD14-FITC, HLA-DR-PerCP, and the corresponding isotype control antibodies. All the antibodies were purchased from BD Pharmingen (San Diego, CA, United States). UC-MSC were analysed via a flow cytometer (BD FACS Calibur™, United States). For functional assays, the PBS-resuspended UC-MSC-EVs preparation was diluted into the corresponding culture medium or zebrafish medium to the indicated final particle concentration. For the HaCaT oxidative-injury viability assays, the UVB-irradiated HSFs viability assay, and the B16F10 melanin content and tyrosinase activity assays, a PBS vehicle control corresponding to the maximum PBS volume introduced with the highest UC-MSC-EVs dose was included.

### 2.2 Cell culture and general experimental methods

#### 2.2.1 Cell lines and culture conditions

HaCaT keratinocytes and B16F10 melanoma cells were purchased from Procell (Wuhan, China), human skin fibroblasts (HSFs) and human dermal papilla cells (hDPCs) were purchased from Shanghai Fuheng Biological Technology Co., Ltd., and SZ95 sebocytes were provided by Henan Academy of Sciences. HaCaT cells, HSFs, and hDPCs were cultured in high-glucose Dulbecco’s modified Eagle’s medium (DMEM) supplemented with 10% fetal bovine serum (FBS) and 1% penicillin-streptomycin (P/S). SZ95 sebocytes were maintained in Sebomed basal medium (Sigma-Aldrich, St. Louis, MO, USA) containing 10% FBS and 5 ng/mL recombinant human epidermal growth factor (Invitrogen, Carlsbad, CA, USA). B16F10 cells were cultured in RPMI-1640 medium supplemented with 10% FBS and 1% P/S. All cells were maintained at 37 °C in a humidified atmosphere containing 5% CO_2_.

#### 2.2.2 Cell viability assay

Cell viability was evaluated using the Cell Counting Kit-8 (CCK-8; Dojindo, Kumamoto, Japan). For viability or cytotoxicity screening, cells were seeded in 96-well plates at the following densities: 1.8 × 10^4^ cells/well for HaCaT cells and 2.0 × 10^4^ cells/well for HSFs, hDPCs, SZ95 sebocytes, and B16F10 cells. After 24 h of attachment, cells were exposed to the treatments specified in the corresponding experimental subsections. At the end of the indicated treatment period, 10 μL of CCK-8 reagent was added to each well and incubated for 1 h. Absorbance at 450 nm was measured using a microplate reader (BioTek, USA).

#### 2.2.3 Immunofluorescence (IF) staining

Immunofluorescence staining was performed on cells grown on glass coverslips. After the designated treatment, cells were fixed with 4% paraformaldehyde (PFA) in PBS for 10–15 min at room temperature. After washing with PBS, cells were permeabilized with 0.5% Triton X-100 in PBS for 15 min at room temperature and blocked with blocking buffer containing 5% bovine serum albumin (BSA) or normal serum in PBS for 1 h at room temperature. Cells were then incubated with target-specific primary antibodies diluted in blocking buffer overnight at 4 °C, followed by incubation with corresponding fluorescent secondary antibodies diluted in blocking buffer for 1 h at room temperature in the dark. Nuclei were counterstained with 1 μg/mL DAPI or Hoechst 33342 (Sigma-Aldrich, US) for 10 min at room temperature. After washing with PBS, coverslips were mounted onto glass slides with anti-fade mounting medium. Images were acquired using an Olympus IX73 fluorescence microscope (Tokyo, Japan). Marker-specific antibodies and treatment conditions are described in the relevant experimental subsections.

#### 2.2.4 Quantitative real-time PCR (qRT-PCR)

Total RNA was extracted from treated cells using an RNeasy mini kit (Qiagen, Shanghai, China) and reverse-transcribed into cDNA using a PrimeScript RT reagent kit (Takara, Dalian, China). qRT-PCR was performed using SYBR Green Premix Ex Taq (Takara, Dalian, China) on a 7500 Real-Time PCR system (Applied Biosystems, USA). Relative gene expression was calculated using the 2^−ΔΔCT^ method with GAPDH as the reference gene. Primer sequences are listed in Supplementary Table S1.

#### 2.2.5 Enzyme-linked immunosorbent assay (ELISA)

Culture supernatants or cell lysates were collected after the designated treatments. Target proteins or metabolites were quantified using analyte-specific commercial ELISA kits according to the manufacturers’ protocols.

#### 2.2.6 Flow cytometry for cell cycle analysis

Cells were harvested, washed with PBS, and fixed in 70% ethanol at 4 °C overnight. Fixed cells were stained with propidium iodide (0.05 mg/mL) in the presence of RNase A (1 mg/mL) and 0.3% Triton X-100 for 30 min in the dark. DNA content was analyzed using a BD FACSCalibur flow cytometer (BD Biosciences, USA), and the proportions of cells in G0/G1, S, and G2/M phases were determined using ModFit LT software (Verity Software House, USA).

### 2.3 Oxidative stress models and antioxidant assays in HaCaT cells

#### 2.3.1 Establishment of oxidative stress models

HaCaT cells were seeded in 96-well plates at 2 × 10^4^ cells/well and allowed to attach for 24 h. For UVB dose screening, the culture medium was replaced with PBS before irradiation, and cells were exposed to 312 nm UVB at 5–50 mJ/cm^2^. After irradiation, PBS was replaced with fresh complete medium and cells were incubated for 24 h before CCK-8 analysis. Based on the preliminary dose-response experiment, 30 mJ/cm^2^ UVB was selected as a sublethal condition that reduced cell viability to approximately 80% of the untreated control (Supplementary Figure S2A).

For the tert-butyl hydroperoxide (tBHP) model, HaCaT cells were exposed to 25–800 μM tBHP in serum-free medium for 24 h. Based on the preliminary dose-response experiment, 100 μM tBHP was selected because it reduced cell viability to approximately 80% of the untreated control while retaining sufficient viable cells for evaluation of UC-MSC-EVs-mediated protection (Supplementary Figure S2B).

#### 2.3.2 UC-MSC-EVs treatment and viability assessment

Immediately after UVB irradiation or tBHP addition, HaCaT cells were treated with UC-MSC-EVs diluted in DMEM containing 10% FBS and incubated for 24 h. Final EVs doses were expressed as particles/mL. Cell viability was determined using the CCK-8 assay as described in Section 2.2.2.

#### 2.3.3 Detection of intracellular reactive oxygen species (ROS) and free radicals

Following oxidative stress induction and EVs treatment, HaCaT cells were incubated with 10 μM 2′,7′-dichlorodihydrofluorescein diacetate (DCFH-DA; Beyotime, Shanghai, China) or 5 μM dihydroethidium (DHE; Beyotime, Shanghai, China) for 30 min at 37 °C in the dark to detect intracellular ROS or superoxide levels, respectively. Fluorescence intensity was observed under a confocal microscope and quantified using ImageJ software (NIH, USA).

#### 2.3.4 Determination of antioxidant enzyme activities

After treatment, HaCaT cells were lysed and the activities of superoxide dismutase (SOD), catalase (CAT), and glutathione peroxidase (GPX) were measured using commercial colorimetric assay kits (Beyotime, Shanghai, China, or Jiancheng Bioengineering Institute, Nanjing, China) according to the manufacturers’ instructions. Enzyme activities were normalized to total protein concentration determined by the BCA assay.

### 2.4 Keratinocyte migration, barrier protein expression, and zebrafish tail-fin regeneration assays

#### 2.4.1 *In vitro* wound-healing assay

HaCaT cells were seeded in 6-well plates at a density of 5 × 10^5^ cells/well and cultured to 90% confluence. A sterile 200 μL pipette tip was used to create a linear scratch. Detached cells were removed by washing with PBS, and the medium was replaced with serum-free DMEM containing designated concentrations of UC-MSC-EVs. Wound closure was photographed at 0, 24, and 48 h using an inverted microscope (Olympus, Tokyo, Japan), and the migration area was quantified using ImageJ software.

#### 2.4.2 Barrier protein expression by immunofluorescence

HaCaT cells were treated with UC-MSC-EVs for 48 h and processed for immunofluorescence staining as described in Section 2.2.3. Rabbit anti-filaggrin (FLG) (1:500; Abcam, ab178435) and rabbit anti-aquaporin-3 (AQP3) (1:500; Abcam, ab125219) antibodies were used to assess barrier- and hydration-related protein expression. Fluorescence intensity was quantified for group comparisons.

#### 2.4.3 Zebrafish tail fin regeneration assay

Zebrafish were maintained in a recirculating aquaculture system (water temperature: 24 °C–28.5 °C; pH: 6.5-7.5; hardness: 6–8 dGH; light/dark cycle: 14/10 h). Healthy embryos at 24 h post-fertilization (hpf) were randomly allocated to 6-well plates and exposed to UC-MSC-EVs at 1.67, 3.33, or 6.67 × 10^8^ particles/mL, with untreated embryos serving as the control. The treatment solution (15 mL/well) was renewed every 24 h, and embryos were maintained at 28 °C ± 0.5 °C. At 72 hpf, caudal fin-fold amputation was performed under a stereomicroscope without damaging the notochord. After amputation, larvae were continuously exposed to the corresponding UC-MSC-EVs concentrations. At 48 and 72 h post-amputation (hpa), three larvae per group were anesthetized with 0.25 mg/mL tricaine (Sigma-Aldrich). Fin-fold regrowth was photographed, and the regenerated area was quantified relative to the amputated area immediately after injury.

### 2.5 UVB-induced senescence and ECM assays in HSFs

#### 2.5.1 Establishment of a UVB-induced photoaging-related fibroblast model

HSFs were seeded in 6-well plates at 4 × 10^5^ cells/well and cultured for 24 h. Cells were washed with PBS and exposed to 100 mJ/cm^2^ UVB to induce photoaging-related cellular changes (Kim et al., 2024). After irradiation, cells were treated with 0.33, 0.67, or 1.33 × 10^8^ particles/mL UC-MSC-EVs for 12h, 24 h, and 48 h. Cell viability was assessed using the CCK-8 assay described in Section 2.2.2. Vitamin C (20 μM) was included as a positive control.

#### 2.5.2 Senescence-associated β-galactosidase (SA-β-Gal) staining

Cellular senescence was evaluated using a SA-β-Gal staining kit (BC2580, Solarbio, Beijing, China). Briefly, treated HSFs were washed with PBS, fixed in SA-β-Gal fixative, and incubated with the staining working solution at 37 °C overnight. Three random fields per slide were imaged under a light microscope, and SA-β-Gal-positive cells were quantified using ImageJ.

#### 2.5.3 ELISA quantification of ECM-related proteins and inflammatory cytokines

The levels of TNF-α, IL-1β, IL-6, type I collagen (Col-I), elastin, and MMP3 were quantified using commercial ELISA kits (Shanghai Enzyme-Linked Biotechnology Co., Ltd., Shanghai, China), and hyaluronic acid (HA) levels were quantified using an ELISA kit from Wuhan Saipei Biotechnology Co., Ltd. (Wuhan, China), according to the manufacturers’ protocols.

#### 2.5.4 COL1A1 and COL3A1 IF staining

Following UVB irradiation and UC-MSC-EVs treatment, HSFs were processed for immunofluorescence staining as described in Section 2.2.3 using primary antibodies against COL1A1 and COL3A1. Fluorescence intensity was quantified for group comparisons.

#### 2.5.5 ECM and antioxidant gene expression

qRT-PCR was performed as described in Section 2.2.4 to quantify ECM-remodeling genes (*COL1A1*, *COL3A1*, Matrix metalloproteinase 1 [*MMP1*], Matrix metalloproteinase 3 [*MMP3*], Matrix metalloproteinase 9 [*MMP9*]), antioxidant genes (heme oxygenase-1 [*HO-1*], NAD(P)H quinone dehydrogenase 1 [*NQO1*], superoxide dismutase 2 [*SOD2*], CAT), and barrier-related genes (*AQP3* and *FLG*).

### 2.6 Oleic acid (OA)-induced lipogenesis and androgen metabolism assays in SZ95 sebocytes

#### 2.6.1 OA-induced excessive lipogenesis model

SZ95 sebocytes were seeded in 96-well plates at 2 × 10^4^ cells/well and cultured for 24 h. When cells reached 80% confluence, oleic acid (OA; Sigma-Aldrich, US) was added to establish the *in vitro* excessive lipogenesis model. Based on dose-response screening, 400 μM OA was selected as the final induction concentration for subsequent experiments (Supplementary Figures S4A–C). UC-MSC-EVs and 10 μM isotretinoin (positive control) were added simultaneously with OA stimulation, and cells were incubated for 48 h. Vehicle-treated cells without OA stimulation served as the blank control.

#### 2.6.2 Intracellular lipid staining and quantification

After 24 h of treatment, cells were washed twice with PBS, fixed with 4% paraformaldehyde for 20 min, and stained with freshly prepared Oil Red O (ORO) working solution (0.5% ORO in isopropanol diluted 3:2 with distilled water, filtered through 0.22 μm) for 30 min. Cells were differentiated in 60% isopropanol for 10 s, washed with PBS, and counterstained with hematoxylin for 5 min. Five random fields per well were imaged under an inverted microscope, and the red-positive area was quantified using Image-Pro Plus software (Media Cybernetics, USA).

For neutral-lipid visualization, cells were stained with Nile Red (5 μg/mL; Sigma-Aldrich) for 15 min at 37 °C and imaged by fluorescence microscopy. Lipid peroxidation was assessed using BODIPY 581/591 C11 (5 μM; Thermo Fisher Scientific) for 30 min, and the fluorescence shift from reduced (red) to oxidized (green) probe was quantified.

#### 2.6.3 Androgen-lipogenic axis analysis

5α-reductase activity in SZ95 sebocytes was measured using a commercially available assay kit (Cayman Chemical, USA) according to the manufacturer’s protocol. Dihydrotestosterone (DHT) levels in cell culture supernatants were quantified by ELISA (Section 2.2.5). Gene expression of SRD5α1 and sterol regulatory element-binding protein 1c (SREBP-1c) was analyzed by qRT-PCR (Section 2.2.4).

### 2.7 Melanogenesis assays in B16F10 cells and zebrafish

#### 2.7.1 α-melanocyte-stimulating hormone (α-MSH)-induced melanogenesis model

B16F10 melanoma cells were seeded at 5 × 10^4^ cells/mL in 6-well plates and cultured for 24 h. Melanogenesis was stimulated with 100 nM α-MSH (Sigma-Aldrich) for 48 h in the presence or absence of UC-MSC-EVs (Kim et al., 2020). Arbutin (1 mM) served as the positive control.

#### 2.7.2 Melanin content and tyrosinase activity assays

Intracellular melanin was extracted using 1 N NaOH at 80 °C for 1 h, and absorbance was measured at 405 nm. Melanin content was normalized to total protein concentration. For tyrosinase activity, cell lysates were incubated with 0.1% L-3,4-dihydroxyphenylalanine (L-DOPA; Sigma-Aldrich) in PBS (pH 6.8) at 37 °C for 1 h, and dopachrome formation was monitored at 475 nm using a microplate reader.

#### 2.7.3 Melanogenic gene expression

qRT-PCR was performed as described in Section 2.2.4 to quantify microphthalmia-associated transcription factor (MITF), tyrosinase (TYR), tyrosinase-related protein 1 (TRP-1) and tyrosinase-related protein 2 (TRP-2).

#### 2.7.4 Zebrafish embryo melanin deposition assay

Synchronized wild-type zebrafish embryos at 6–8 hpf were randomly distributed into 6-well plates (30 embryos per well) containing E3 embryo medium. Embryos were exposed to 1.67 × 10^8^, 3.33 × 10^8^, or 6.67 × 10^8^ particles/mL UC-MSC-EVs. Fresh test medium was renewed every 24 h at 28 °C ± 0.5 °C. At 48 and 72 hpf, larvae were anesthetized with MS-222 solution, mounted in 3% methylcellulose for consistent orientation, and imaged using an Olympus SZX16 stereomicroscope (Tokyo, Japan). Melanin pigment intensity of the dorsal trunk region was quantified by ImageJ software. Untreated embryos incubated in E3 medium were set as the blank control.

### 2.8 hDPCs functional and androgen metabolism assays

#### 2.8.1 hDPCs proliferation and migration

hDPCs were seeded in 96-well plates, and cell viability was assessed by CCK-8 (Section 2.2.2) after 48 h of UC-MSC-EVs treatment. For migration assessment, hDPCs were seeded in 6-well plates, and a linear scratch was created as described in Section 2.4.1. Wound closure was monitored at 0 h and 24 h.

#### 2.8.2 Alkaline phosphatase (ALP) activity

hDPCs were treated with UC-MSC-EVs for 48 h, and ALP activity was measured using a colorimetric assay kit (Beyotime, Shanghai, China) with p-nitrophenyl phosphate (pNPP) as the substrate. Absorbance was measured at 405 nm, and activity was normalized to total protein content.

#### 2.8.3 Androgen metabolism and growth factor expression

5α-reductase activity in hDPCs was measured as described in Section 2.6.3. DHT levels were quantified by ELISA (Section 2.2.5). Gene expression of SRD5α1, vascular endothelial growth factor (VEGF), insulin-like growth factor-1 (IGF-1), and fibroblast growth factor-7 (FGF-7) was analyzed by qRT-PCR (Section 2.2.4).

#### 2.8.4 Cell cycle analysis

After 48 h of UC-MSC-EVs treatment, hDPC cell-cycle distribution was analyzed by propidium iodide staining and flow cytometry as described in Section 2.2.6.

### 2.9 Statistical analysis

All experiments were independently repeated three times. Technical replicate wells were included within each independent experiment as indicated by the experimental design. Technical replicates were averaged within each independent experiment, and the resulting values from three independent experiments were used for statistical analysis. Data are presented as mean ± standard deviation (SD). Statistical analyses were performed using GraphPad Prism 9.3.0 (GraphPad Software, San Diego, CA, USA). Two-group comparisons were performed using unpaired two-tailed Student’s t-tests. For experiments with more than two groups, one-way analysis of variance (ANOVA) followed by Tukey’s multiple-comparison test was used. Exact sample sizes are reported in the corresponding figure legends. *P* < 0.05 was considered statistically significant.

## 3 Results

### 3.1 Characterization of UC-MSC-EVs

UC-MSC-EVs were isolated using a production strategy combining microcarrier-based culture with extracellular vesicle harvesting to enable large-scale preparation. The particle-to-protein ratio of the UC-MSC-EVs preparation was 1.11 × 10^8^ particles/µg. TEM revealed that the UC-MSC-EVs had a typical saucer-like morphology, with a size of approximately 100 nm (Supplementary Figure S1A). NTA was performed with a NanoSight instrument to measure the diameter of the extracellular vesicles, which had an average diameter of 100 nm (Supplementary Figures S1B,C). Moreover, Western blot analysis confirmed the enrichment of canonical extracellular vesicle markers, including CD9, CD63, Tsg101, and Alix, whereas the negative markers GM130 and Calnexin were not detected (Supplementary Figure S1D). Flow-cytometric analysis showed that the UC-MSCs used for EVs production were positive for CD90, CD73, and CD105, with CD29 also highly expressed, whereas CD79a, CD34, CD45, HLA-DR, and CD14 showed negative expression (Supplementary Figure S1E). These results demonstrated the successful isolation and characterization of UC-MSC-EVs for subsequent functional analyses.

### 3.2 UC-MSC-EVs attenuate oxidative injury in keratinocytes

To determine whether UC-MSC-EVs exert cytoprotective effects under oxidative injury, we first evaluated their effects on HaCaT cell viability and found no significant reduction in viability within the tested concentration range (Figure 1A). This concentration range was therefore used for subsequent functional analyses.

**FIGURE 1:**
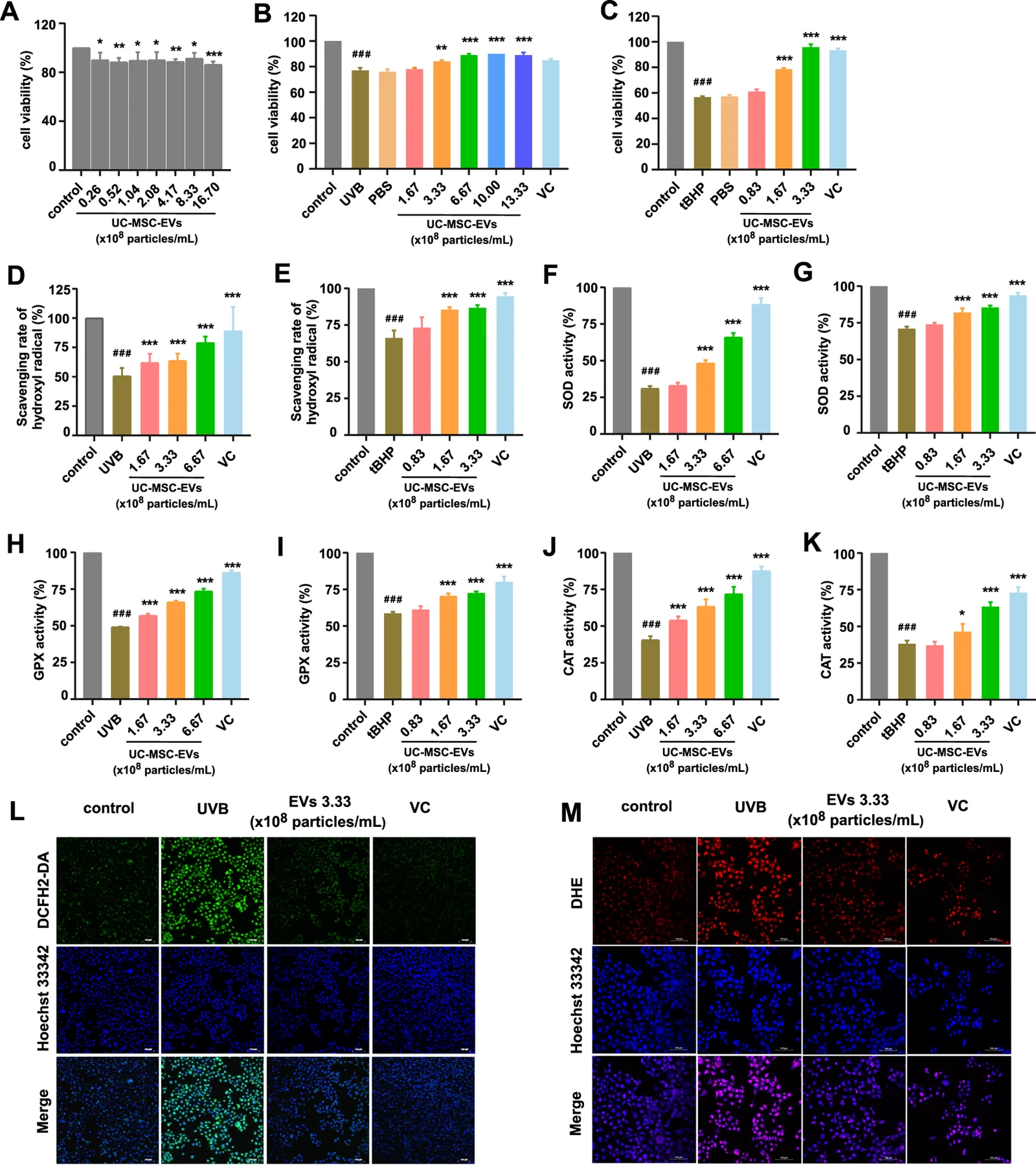
UC-MSC-EVs attenuate oxidative injury and restore antioxidant defense in HaCaT keratinocytes. **(A)** Relative viability of HaCaT cells treated with UC-MSC-EVs, as determined using the CCK-8 assay. Cell viability in the untreated control group was normalized to 100%. **(B,C)** Effects of UC-MSC-EVs on the viability of HaCaT cells exposed to UVB irradiation **(B)** or tert-butyl hydroperoxide (tBHP) **(C)**. Following induction of oxidative injury, cells were treated with increasing concentrations of UC-MSC-EVs. **(D,E)** Effects of UC-MSC-EVs on hydroxyl radical accumulation in HaCaT cells exposed to UVB irradiation **(D)** or tBHP **(E)**. **(F,G)** Effects of UC-MSC-EVs treatment on SOD activity in HaCaT cells subjected to UVB-induced **(F)** or tBHP-induced **(G)** oxidative stress. **(H,I)** Effects of UC-MSC-EVs treatment on GPX activity in HaCaT cells subjected to UVB **(H)** or tBHP-induced **(I)** oxidative stress. **(J,K)** Effects of UC-MSC-EVs treatment on CAT activity in HaCaT cells subjected to UVB **(J)** or tBHP-induced **(K)** oxidative stress. **(L)** Effects of UC-MSC-EVs on intracellular reactive oxygen species levels in UVB-irradiated HaCaT cells. **(M)** Effects of UC-MSC-EVs on superoxide anion levels in UVB-irradiated HaCaT cells. ^#^ *P* < 0.05, ^##^ *P* < 0.01, ^###^ *P* < 0.001 versus the untreated control group; *P < 0.05, **P < 0.01, ***P < 0.001 versus the corresponding UVB- or tBHP-treated model group.

Two complementary oxidative-stress models were then established using UVB irradiation and tBHP exposure. Preliminary CCK-8 dose-response experiments identified 30 mJ/cm^2^ UVB and 100 µM tBHP as sub-lethal conditions that produced measurable injury while retaining sufficient viable cells for assessment of protection (Supplementary Figures S2A,B). Under both conditions, cell viability was significantly reduced, whereas UC-MSC-EVs treatment significantly increased viability relative to the corresponding injured model groups (Figures 1B,C). UVB and tBHP also increased hydroxyl radical accumulation, which was markedly attenuated by EVs treatment (Figures 1D,E).

Exposure to UVB and tBHP also decreased the activities of the endogenous antioxidant enzymes SOD, GPX, and CAT, whereas UC-MSC-EVs treatment significantly restored these antioxidant defenses (Figures 1F–K).

In UVB-irradiated HaCaT cells, fluorescence-based assays further showed lower intracellular ROS accumulation and reduced superoxide signals after UC-MSC-EVs treatment (Figures 1L,M). Together, the concordant changes demonstrate antioxidant and cytoprotective effects of UC-MSC-EVs in oxidatively stressed keratinocytes.

### 3.3 UC-MSC-EVs promote keratinocyte repair and zebrafish tail-fin regeneration

We next examined cellular processes relevant to epidermal repair. In UVB-injured HaCaT cells, UC-MSC-EVs treatment promoted cellular recovery in a dose-dependent manner, with the largest effect observed at 6.67 × 10^7^ particles/mL after 48 h (Figure 2A). In wound-healing assays, 8.33 × 10^7^ particles/mL UC-MSC-EVs increased wound closure at both 24 h and 48 h (Figures 2B,C). UC-MSC-EVs treatment also increased FLG and AQP3 fluorescence relative to the control group (Figures 2D,E). FLG is associated with epidermal differentiation and barrier function, whereas AQP3 mediates water and glycerol transport in the epidermis.

**FIGURE 2:**
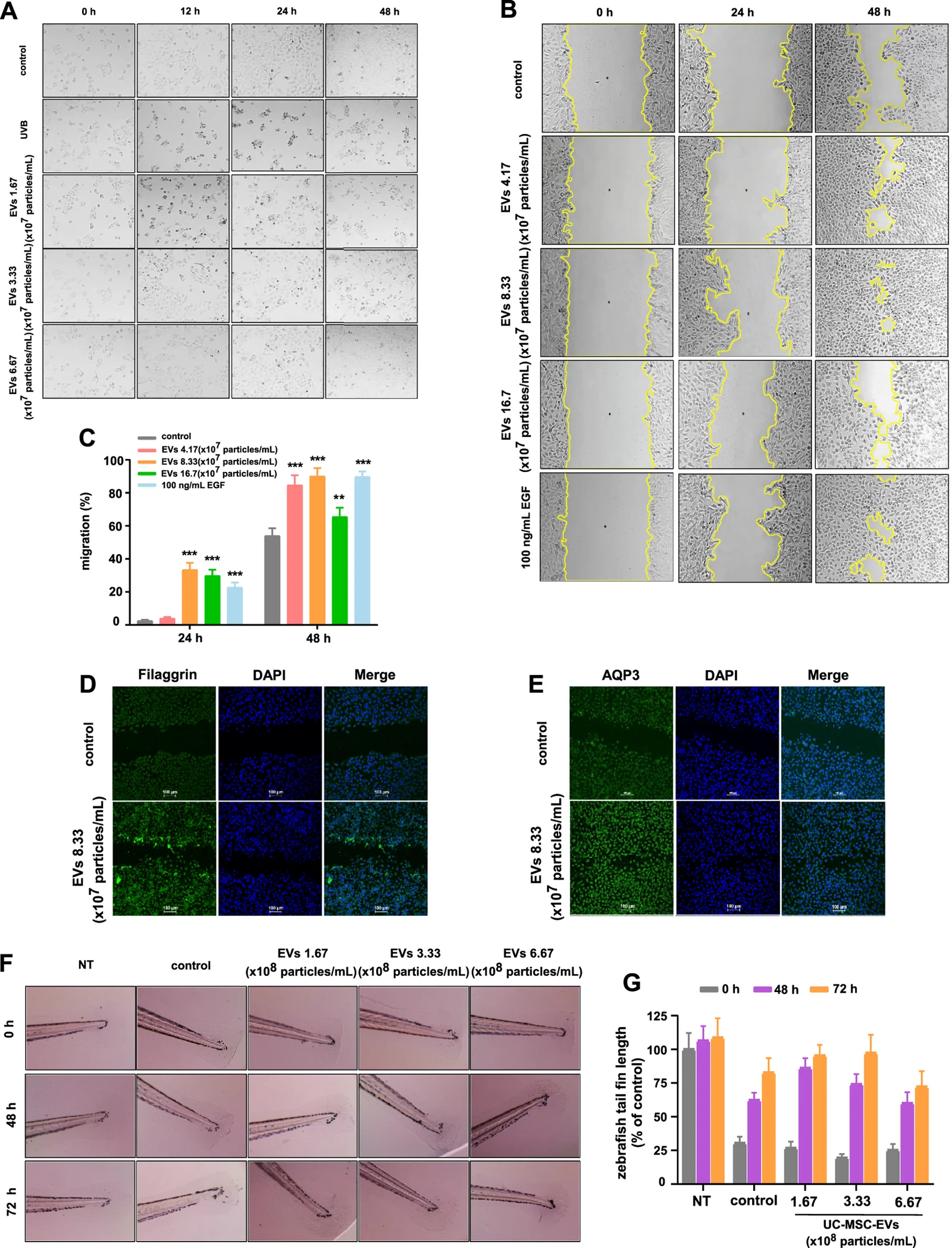
UC-MSC-EVs promote keratinocyte repair and zebrafish tail-fin regeneration. **(A)** Effects of UC-MSC-EVs on the recovery of UVB-injured HaCaT cells. HaCaT cells were exposed to UVB irradiation and subsequently treated with increasing concentrations of UC-MSC-EVs for 12, 24, or 48 h. Cell recovery was evaluated at the indicated time points. **(B)** Representative images of the scratch wound-healing assay in HaCaT cells treated with different concentrations of UC-MSC-EVs for 0, 24, and 48 h. **(C)** Quantification of HaCaT cell migration in the scratch wound-healing assay. **(D,E)** Representative immunofluorescence images and quantitative analysis of filaggrin **(D)** and AQP3 **(E)** expression in HaCaT cells following UC-MSC-EVs treatment. **(F)** Representative images of zebrafish caudal fin regeneration following amputation and treatment with different concentrations of UC-MSC-EVs. **(G)** Quantification of the caudal fin regeneration rate at the indicated time points after amputation. *P < 0.05, **P < 0.01, and ***P < 0.001 versus the corresponding control group.

The zebrafish caudal fin regeneration assay was used as an *in vivo* model of tissue repair to complement the keratinocyte migration assay. This model allowed us to assess whether the repair-promoting effects of UC-MSC-EVs observed *in vitro* could also be detected at the tissue level *in vivo*. In the zebrafish tail-fin amputation model, the relative regenerated length of the model group reached 60.24% ± 4.01% at 48 h and 80.41% ± 5.47% at 72 h, normalized to uninjured controls. Treatment with UC-MSC-EVs markedly promoted fin regrowth, with the 1.67 × 10^8^ particles/mL group showing the most pronounced enhancement, achieving 84.10% ± 5.82% relative regenerated length at 48 h and 93.72% ± 6.30% at 72 h (Figures 2F,G).

### 3.4 UC-MSC-EVs attenuate UVB-induced photoaging-associated changes in HSFs

To investigate the effects of UC-MSC-EVs on UVB-induced photoaging-associated changes in HSFs, their cytotoxicity was first assessed. The results showed that UC-MSC-EVs at concentrations of 0.33, 0.67, and 1.33 × 10^8^ particles/mL were well tolerated by HSFs and were therefore selected for subsequent experiments (Figures 3A,B). UVB irradiation markedly reduced cell viability and increased the number of SA-β-Gal-positive senescent cells, whereas UC-MSC-EVs treatment significantly improved cell viability and reduced cellular senescence. The 0.33 × 10^8^ particles/mL group showed the strongest effect, exceeding that observed with vitamin C (Figures 3C,D).

**FIGURE 3:**
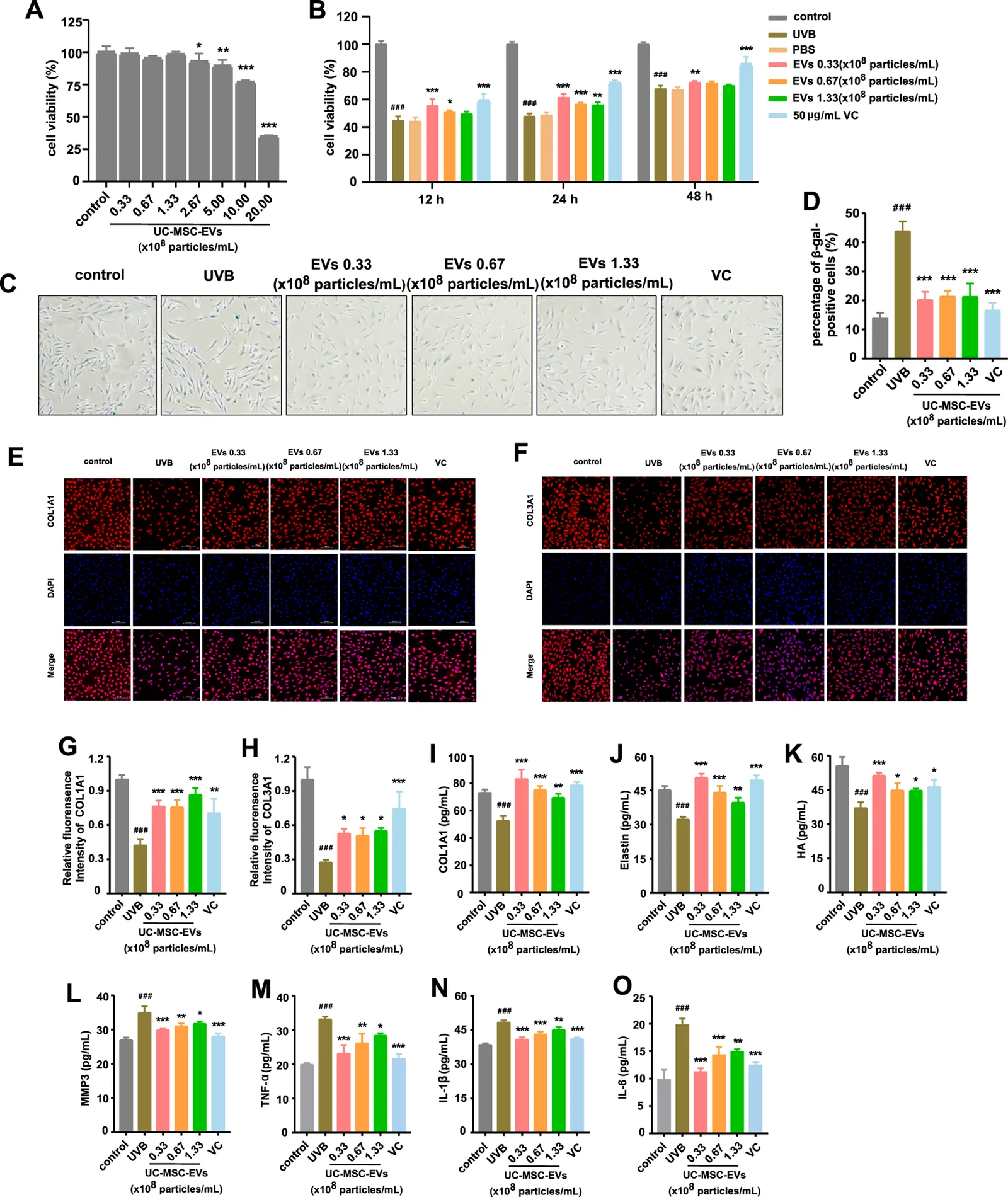
UC-MSC-EVs attenuate UVB-induced senescence and ECM alterations in human skin fibroblasts. **(A)** Relative viability of HSFs treated with increasing concentrations of UC-MSC-EVs, as determined using the CCK-8 assay. Cell viability in the untreated control group was normalized to 100%. **(B)** Effects of UC-MSC-EVs on the viability of UVB-irradiated HSFs. Following UVB irradiation, cells were treated with increasing concentrations of UC-MSC-EVs. **(C)** Representative images of cellular senescence-associated β-galactosidase (SA-β-Gal) staining in UVB-irradiated HSFs treated with different concentrations of UC-MSC-EVs. Blue-stained cells were considered SA-β-Gal-positive senescent cells. **(D)** Quantification of SA-β-Gal-positive cells. **(E,F)** Representative immunofluorescence images of type I collagen (COL1A1) **(E)** and type III collagen (COL3A1) **(F)** in UVB-irradiated HSFs treated with UC-MSC-EVs at concentrations of 0.33 × 10^8^, 0.67 × 10^8^, and 1.33 × 10^8^ particles/mL. Scale bar = 50 μm. **(G,H)** Quantification of COL1A1 **(G)** and COL3A1 **(H)** immunofluorescence signals. **(I–L)** Quantification of COL1A1 **(I)**, elastin **(J)**, hyaluronic acid (HA) **(K)**, and matrix metalloproteinase-3 (MMP-3) **(L)** levels in UVB-irradiated HSFs following UC-MSC-EVs treatment. **(M–O)** Quantification of TNF-α **(M)**, IL-1β **(N)**, and IL-6 **(O)** levels in UVB-irradiated HSFs following UC-MSC-EVs treatment. ^#^ *P* < 0.05, ^##^ *P* < 0.01, ^###^ *P* < 0.001 versus the control group; ^*^ *P* < 0.05, ^**^ *P* < 0.01, ^***^ *P* < 0.001 versus the UVB model group.

Because ECM degradation is a major feature of UVB-induced fibroblast dysfunction, we next examined ECM protein changes following UC-MSC-EVs treatment. Immunofluorescence staining showed that UVB exposure markedly reduced COL1A1 and COL3A1 protein expression, whereas UC-MSC-EVs treatment restored collagen I and collagen III fluorescence intensity in HSFs (Figures 3E–H). Consistently, ELISA analysis demonstrated that UC-MSC-EVs increased the levels of COL1A1, elastin, and hyaluronic acid, while reducing MMP3 levels in UVB-irradiated HSFs, and these changes showed similar trends to those observed in the Vtamin C-positive control group (Figures 3I–L).

UVB exposure also induced an inflammatory response, as indicated by elevated secretion of TNF-α, IL-1β, and IL-6. Treatment with UC-MSC-EVs significantly reduced the levels of these pro-inflammatory cytokines, and the Vtamin C positive control produced a similar inhibitory response (Figures 3M–O).

To further examine transcriptional changes, qRT-PCR analysis was performed (Supplementary Figure S3). UVB irradiation altered the expression of genes involved in ECM remodeling, antioxidant defense, and barrier function, including COL1A1, COL3A1, Elastin, MMP1, MMP3, MMP9, HO-1, NQO1, SOD2, CAT, AQP3, and FLG. UC-MSC-EVs treatment partially or markedly reversed these UVB-induced transcriptional changes, broadly consistent with the protein-level and cellular responses observed in Figure 3.

### 3.5 UC-MSC-EVs suppress OA-induced lipogenesis, lipid peroxidation, and androgenic responses in SZ95 sebocytes

To assess the effects of UC-MSC-EVs on sebocyte lipid metabolism, an OA-induced lipogenesis model was established in SZ95 sebocytes. UC-MSC-EVs showed no obvious cytotoxicity at concentrations ≤1.33 × 10^7^ particles/mL, which were therefore used for subsequent assays (Figure 4A). ORO and Nile Red staining revealed that UC-MSC-EVs significantly attenuated OA-induced lipid deposition and lipid droplet accumulation, with changes occurring in the same direction as those observed with 10 μM isotretinoin (Figures 4B–D). Moreover, the results of BODIPY 581/591 C11 staining indicated that OA exposure also increased lipid peroxidation, whereas UC-MSC-EVs significantly reduced the oxidized BODIPY signal (green) (Figures 4E,F). 10 μM isotretinoin also suppressed OA-induced lipid peroxidation.

**FIGURE 4:**
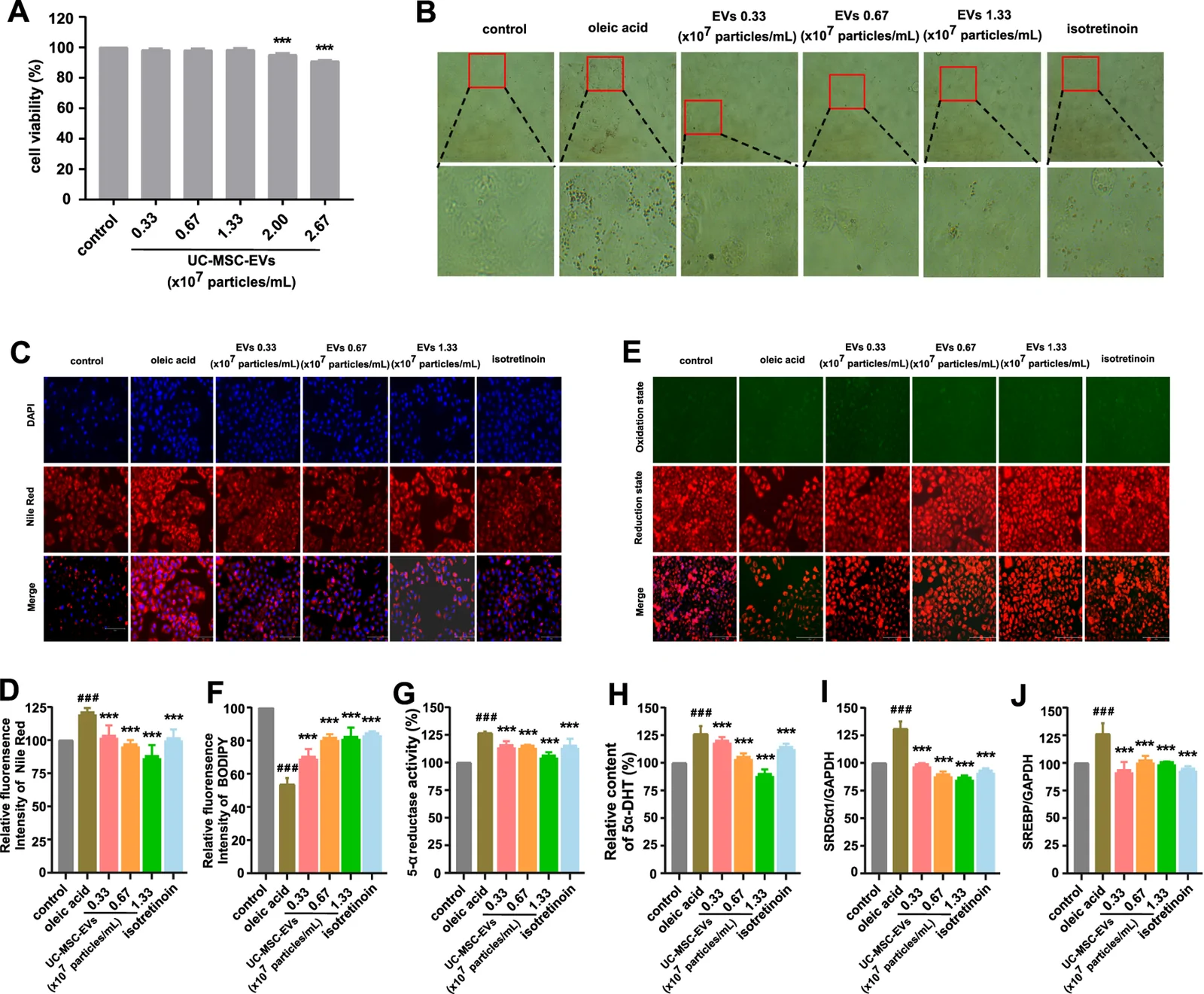
UC-MSC-EVs suppress OA-induced lipogenesis, lipid peroxidation, and androgen metabolism in SZ95 sebocytes. **(A)** Effects of increasing concentrations of UC-MSC-EVs on the viability of SZ95 sebocytes. **(B,C)** Representative images of Oil Red O staining **(B)** and Nile Red staining **(C)** in oleic acid (OA)-stimulated SZ95 sebocytes treated with different concentrations of UC-MSC-EVs. **(D)** Quantification of the relative fluorescence intensity of Nile Red staining. **(E)** Representative images of BODIPY 581/591 C11 staining in OA-stimulated SZ95 sebocytes treated with different concentrations of UC-MSC-EVs. Scale bar = 20 μm. **(F)** Quantification of the relative fluorescence intensity of the BODIPY 581/591 C11 probe. **(G)** Effects of UC-MSC-EVs on 5α-reductase activity in SZ95 sebocytes following 12 h of OA stimulation. **(H)** Effects of UC-MSC-EVs on relative DHT levels in SZ95 sebocytes following 12 h of OA stimulation. **(I,J)** Relative mRNA expression levels of SRD5A1 **(I)** and SREBP-1c **(J)**, as determined by qRT-PCR ^#^ *P* < 0.05, ^##^ *P* < 0.01, and ^###^ *P*< 0.001 versus the untreated control group; ^*^ *P* < 0.05, ^**^ *P* < 0.01, and ^***^ *P* < 0.001 versus the OA-treated model group.

OA stimulation increased 5α-reductase activity and DHT production in SZ95 sebocytes, whereas UC-MSC-EVs treatment significantly reduced both readouts (Figures 4G,H). SRD5A1 and SREBP-1c expression were also decreased after EVs treatment (Figures 4I,J). The isotretinoin group showed the expected inhibitory effects on these androgenic and lipogenic responses. These results demonstrate that UC-MSC-EVs suppress sebocyte lipogenesis and lipid peroxidation, with concurrent downregulation of androgen-associated and SREBP-1c-related lipogenic responses.

### 3.6 UC-MSC-EVs inhibit melanogenesis in B16F10 cells and reduce pigmentation in zebrafish

We further evaluated the effects of UC-MSC-EVs on melanogenesis in B16F10 melanocytes, given that UV-associated oxidative stress can contribute to abnormal melanogenic responses. Based on B16F10 cell tolerability, doses of 0.33, 0.67, and 1.33 × 10^8^ particles/mL were used for functional assays (Figure 5A). In 100 nM α-MSH-stimulated B16F10 cells, melanin content was elevated to 147.60% ± 15.98% of the control level, whereas UC-MSC-EVs at highest dose reversed it to 99.70% ± 7.98% (Figure 5B). Concurrently, tyrosinase activity, which was significantly upregulated by α-MSH, was dose-dependently suppressed by UC-MSC-EVs, reaching 33.53% ± 2.19% at the maximum concentration (Figure 5C). Notably, the inhibitory effects at this dose were comparable to those of the 1 mM arbutin positive control. At the transcriptional level, α-MSH stimulation upregulated the expression of MITF, TYR, TRP-1 and TRP-2, whereas UC-MSC-EVs treatment dose-dependently reduced their expression (Figures 5D–G).

**FIGURE 5:**
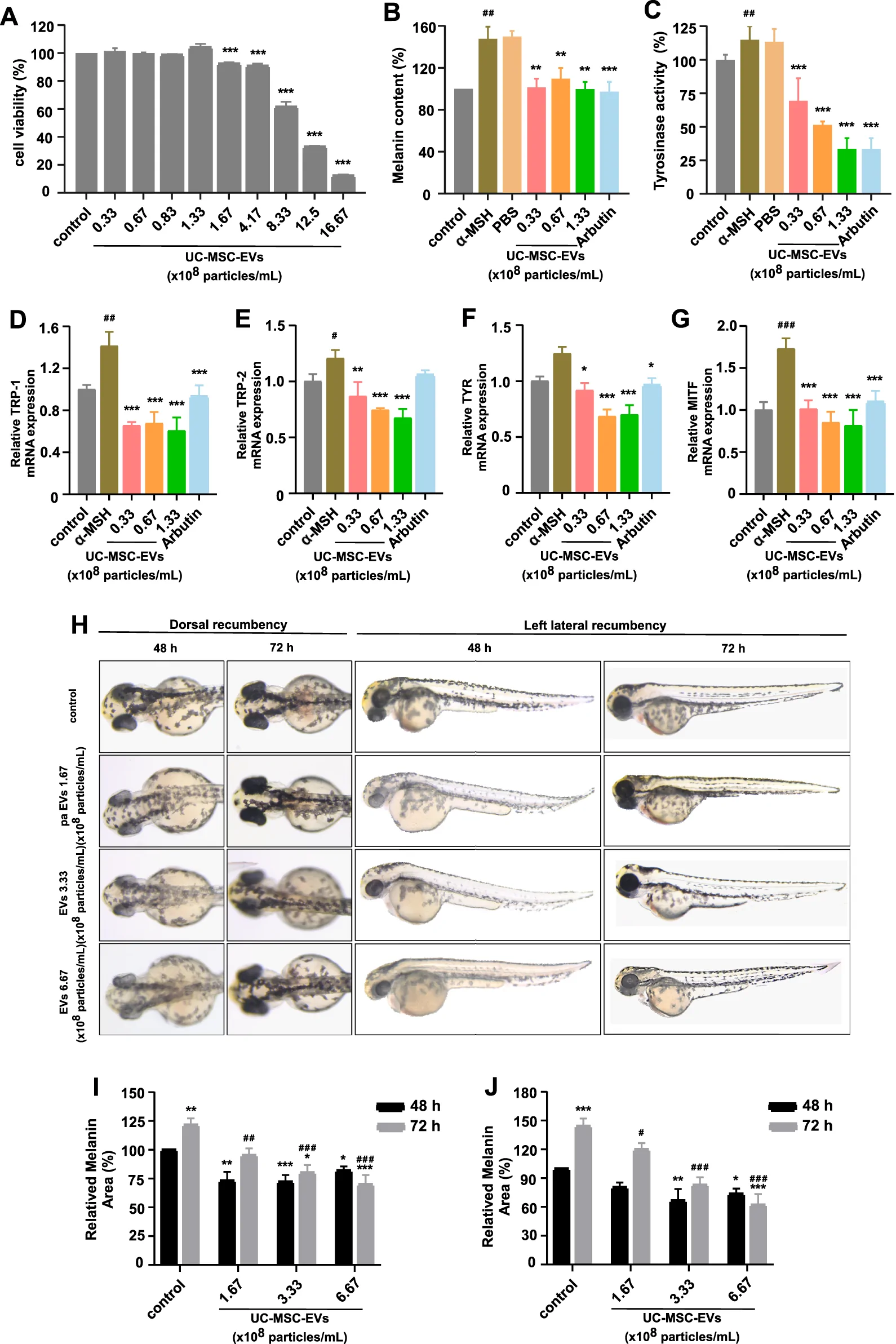
UC-MSC-EVs inhibit melanogenesis in B16F10 cells and reduce pigmentation in zebrafish. **(A)** Effects of increasing concentrations of UC-MSC-EVs on the viability of B16F10 cells. **(B,C)** Effects of UC-MSC-EVs on intracellular melanin content **(B)** and tyrosinase activity **(C)** in α-MSH-stimulated B16F10 cells. **(D–G)** Relative mRNA expression levels of TRP-1 **(D)**, TRP-2 **(E)**, TYR **(F)**, and MITF **(G)**, as determined by qRT-PCR. **(H)** Representative bright-field images of zebrafish larvae showing pigmentation changes under UC-MSC-EVs treatment. **(I,J)** Quantification of relative melanin area from dorsal **(I)** and left-lateral **(J)** views. Data are presented as mean ± SD. ^#^ *P* < 0.05, ^##^ *P* < 0.01, and ^###^ *P* < 0.001 versus the untreated control group; ^*^ *P* < 0.05, ^**^ *P* < 0.01, and ^***^ *P* < 0.001 versus the model group.

UC-MSC-EVs also reduced melanin intensity in zebrafish (Figure 5H). ImageJ quantification demonstrated that UC-MSC-EVs treatment reduced zebrafish pigmentation relative to the 48-h baseline. Dose-dependent depigmenting effects were observed in both dorsal and lateral views. The most evident effect was observed at 6.67 × 10^8^ particles/mL after 72 h of treatment, with melanin intensity reduced to 70.05% ± 8.0% (dorsal) and 62.31% ± 11.1% (lateral) relative to the control (Figures 5I,J). Together, the cellular and zebrafish results demonstrate consistent inhibitory effects of UC-MSC-EVs on melanogenesis.

### 3.7 UC-MSC-EVs enhance hDPC function and reduce androgen metabolism

To investigate the hair growth-related potential of UC-MSC-EVs, we assessed their effects on hDPCs. We first determined that UC-MSC-EVs concentrations up to 1.33 × 10^7^ particles/mL did not compromise hDPC viability and thus used this range for subsequent assays (Figure 6A). UC-MSC-EVs treatment significantly increased scratch closure (Figures 6B,C). UC-MSC-EVs treatment also increased ALP activity, with the strongest effect observed at 0.67 × 10^7^ particles/mL (approximately 148.22% ± 4.72% of the control; Figure 6D). In parallel, 5α-reductase activity was dose-dependently reduced, with the greatest suppression at 1.33 × 10^7^ particles/mL (approximately 86.71% ± 1.41% of the control; Figure 6E). These results indicate enhanced dermal papilla cell functional activity accompanied by attenuated androgen metabolism *in vitro*.

**FIGURE 6:**
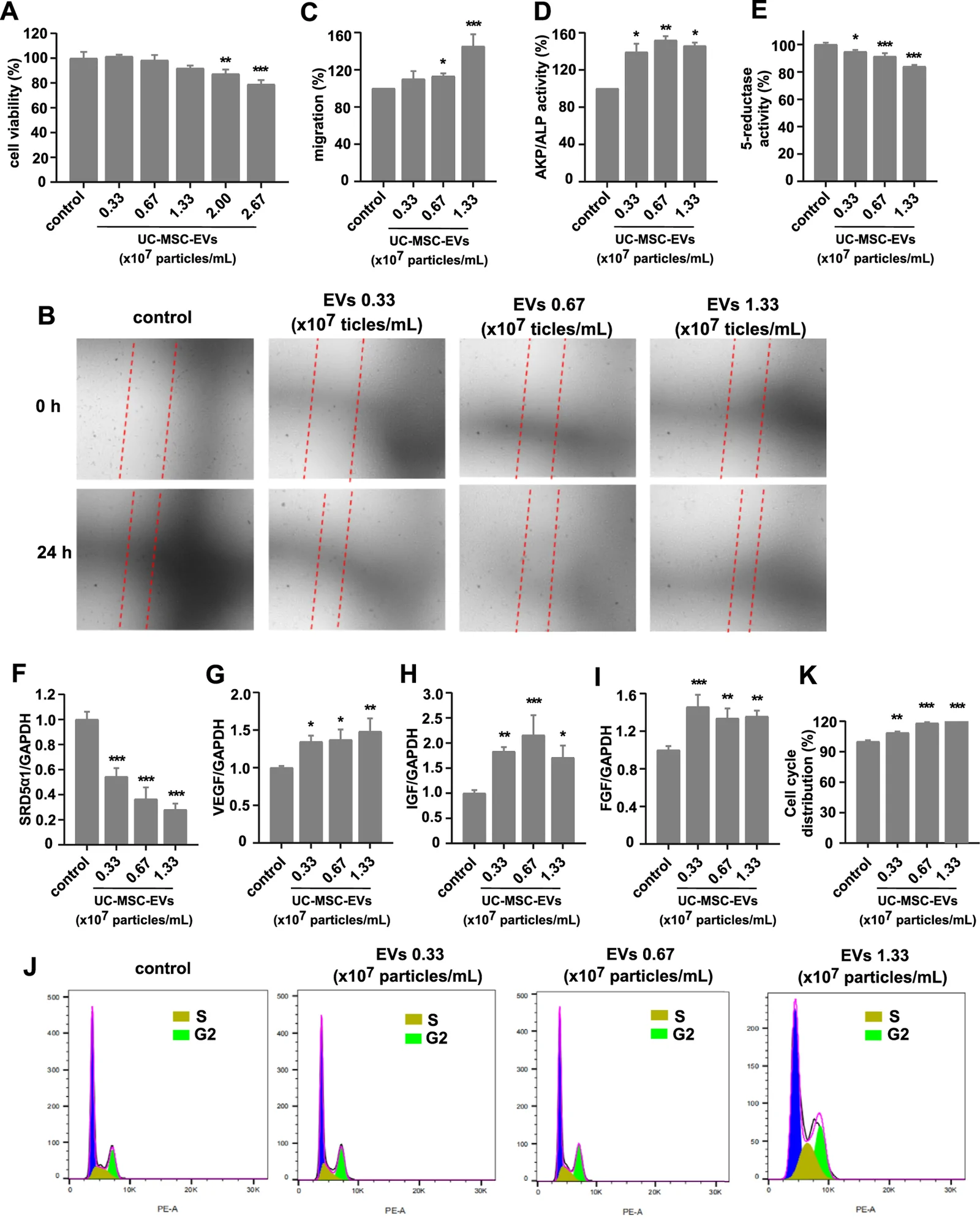
UC-MSC-EVs enhance hDPC functional activity and reduce androgen metabolism. **(A)** Effects of increasing concentrations of UC-MSC-EVs on hDPC viability. **(B)** Representative images of the scratch wound-healing assay in hDPCs treated with different concentrations of UC-MSC-EVs at 0 and 24 h. **(C)** Quantification of hDPC migration. **(D)** Effects of UC-MSC-EVs on ALP activity. **(E)** Effects of UC-MSC-EVs on 5α-reductase activity. **(F–I)** Relative mRNA expression levels of SRD5A1 **(F)**, VEGF **(G)**, IGF-1 **(H)**, and FGF-7 **(I)**, as determined by qRT-PCR. **(J,K)** Cell-cycle distribution of hDPCs following UC-MSC-EVs treatment, as determined by flow cytometry. Representative profiles are shown in **(J)**, and the percentages of cells in the G0/G1, S, and G2/M phases are quantified in **(K)**. *P < 0.05, **P < 0.01, and ***P < 0.001 versus the untreated control group.

UC-MSC-EVs decreased SRD5A1 expression while increasing VEGF, IGF-1, and FGF-7 expression (Figures 6F–I). Flow-cytometric analysis further showed a dose-dependent redistribution of hDPCs toward S and G2/M phases (Figures 6J,K), consistent with increased cell-cycle activity. These molecular and cellular changes are relevant to dermal papilla function, follicular growth support, and androgen responsiveness. Together, these findings show that UC-MSC-EVs influence multiple functional features of hDPCs, including migration, ALP activity, growth factor expression, cell-cycle distribution, and androgen metabolism.

## 4 Discussion

The present study provides a cross-model assessment of the biological activity of a single UC-MSC-EVs preparation. This is relevant because published skin-related MSC-EV studies differ substantially in MSC source, EVs preparation, dose, recipient cell type, and experimental conditions, making biological effects difficult to compare directly across studies. Recent studies have begun to evaluate MSC-EVs across more than one skin-related endpoint. For example, Zhang et al. assessed adipose- and umbilical cord-derived MSC-EVs in keratinocytes, fibroblasts, reconstructed skin, and animal photoaging models (Zhang H. et al., 2024), whereas Kee et al. examined UC-MSC-derived small EVs in relation to fibroblast ECM regulation and melanogenesis (Kee et al., 2025). The present study extends this work by applying the same UC-MSC-EVs preparation across a broader set of complementary experimental systems and by including sebocyte lipid metabolism, lipid peroxidation, and androgen metabolism, which remain comparatively less studied. Together, these findings provide a cross-model biological activity profile of a single UC-MSC-EVs preparation across multiple skin-related processes.

UV-induced oxidative stress is a major driver of cellular senescence, inflammation, and ECM degradation in skin (Fisher et al., 2002; Pillai et al., 2005). The responses observed in keratinocytes and fibroblasts are broadly consistent with the growing evidence that MSC-EVs can modify cellular responses to UV and oxidative stress. Zhang et al. reported that both adipose- and umbilical cord-derived MSC-EVs reduced UVB-induced ROS accumulation, senescence, inflammation, and ECM alterations in keratinocytes, dermal fibroblasts, and reconstructed skin, and further identified TIMP1/Notch1 signaling as a contributing mechanism (Zhang H. et al., 2024). Sun et al. similarly showed that UC-MSC-derived small EVs reduced oxidative stress and collagen degradation in UV-exposed dermal fibroblasts, with pregnancy zone protein linked to regulation of MMP-1 and DNA damage (Sun et al., 2024). These mechanistic studies provide useful context for the antioxidant effects, cellular senescence, and ECM changes observed here. Our study extends these observations by evaluating stress responses together with keratinocyte migration and the barrier markers FLG and AQP3, which have established roles in epidermal barrier function and hydration (Sandilands et al., 2009; Bollag et al., 2020). The zebrafish caudal-fin assay, which captures tissue-scale dynamics during wound healing and regeneration (Tan et al., 2025; Gemberling et al., 2013), provided tissue-level evidence of repair activity complementary to the cell-based migration assays.

Sebaceous gland homeostasis depends on tightly regulated lipid synthesis and androgenic control (Clayton et al., 2019; Del Rosso and Kircik, 2024). A less extensively studied finding was the effect of UC-MSC-EVs on sebocyte lipogenesis and lipid peroxidation in SZ95 sebocytes. Zhang et al. recently reported that adipose-derived MSC vesicles promoted SZ95 sebocyte proliferation and regulated SREBP-1 and lipid accumulation through PI3K/AKT signaling in a wound-healing context, with the response influenced by oxygen conditions (Zhang Y. et al., 2024). In contrast, under the OA-induced lipogenic conditions used in our study, UC-MSC-EVs treatment reduced lipid accumulation and lipid peroxidation and was accompanied by lower SREBP-1c expression. In addition to using different MSC sources, the two studies addressed distinct sebocyte conditions: sebocyte activation upon tissue repair and OA-induced excessive lipid accumulation. The present study further extends published sebocyte work by examining androgen metabolism, including 5α-reductase activity, DHT production, and SRD5A1 expression. The different responses observed between the two studies may be related to differences in EVs source and sebocyte stimulation conditions. Our findings extend previous sebocyte studies by showing that UC-MSC-EVs also reduce lipid peroxidation and influence androgen metabolism in sebocytes.

Pigmentation is a major skin-related process influenced by oxidative and cellular stress (Slominski et al., 2004). Recent studies have shown that UC-MSC-derived small EVs reduced melanin synthesis in MNT-1 melanoma cells, although tyrosinase activity was not significantly altered (Kee et al., 2025). By comparison, the present study showed concurrent decreases in melanin content, tyrosinase activity, and MITF, TYR, TRP-1, and TRP-2 expression in α-MSH-stimulated B16F10 cells. This pattern is closer to the findings of Lee et al., who reported reductions in melanin content, tyrosinase activity, and melanogenic markers following treatment of B16F10 cells with fibroblast-derived EVs (Lee et al., 2024). Importantly, EVs effects on pigmentation are not uniformly inhibitory. Wang et al. found that MSC-derived vesicles promoted melanogenesis in primary melanocytes exposed to H_2_O_2_ while protecting the cells from oxidative injury (Wang et al., 2024). The different directions of these responses suggest that EVs regulation of pigmentation is strongly influenced by recipient-cell state, experimental stimulus, EVs source, and dose. In the present study, the reduction in zebrafish pigmentation provided organism-level support for the anti-melanogenic effects observed in the α-MSH-stimulated B16F10 model.

Dermal papilla cells are key regulators of hair follicle development and cycling (Paus and Cotsarelis, 1999; Schneider et al., 2009), and both growth factor signaling and androgen metabolism are important determinants of their function in androgenetic alopecia (Inui and Itami, 2013; Kwack et al., 2008). Our findings are broadly consistent with recent studies of UC-MSC-derived EVs in hDPCs. Chen et al. showed that human UC-MSC-derived vesicles enhanced hDPC growth and increased the proportion of cells in S and G2/M phases, with PI3K/Akt/GSK-3β signaling implicated in the proliferative response (Chen et al., 2025). The similar cell-cycle redistribution observed in our study supports this growth-related effect, while the changes in migration, ALP activity, VEGF, IGF-1, and FGF-7 extend the comparison to other aspects of hDPC function. More recently, Yu et al. demonstrated hair-follicle enlargement and anagen-associated responses in a DHT-induced androgenetic alopecia mouse model and linked these effects to EVs-enriched miRNAs and Wnt/β-catenin signaling (Yu et al., 2025). In contrast to that *in vivo* study, the present work specifically examined androgen metabolism within cultured hDPCs and found reduced 5α-reductase activity and SRD5A1 expression in parallel with growth-supportive responses. Notably, our study examined growth-supportive functions and androgen metabolism within the same hDPC system, adding an androgen-regulatory dimension to previous reports of UC-MSC-EVs activity in dermal papilla cells.

In summary, this study defines a multi-model biological activity profile of a characterized UC-MSC-EVs preparation across several skin-related processes. In addition to the previously reported effects of MSC-EVs on oxidative stress, epidermal repair, and ECM regulation, our findings extend their biological activity to sebocyte lipogenesis, lipid peroxidation, androgen metabolism, melanogenesis, and dermal papilla cell function. The sebocyte results are particularly notable because lipid peroxidation and androgen metabolism have received comparatively limited attention in previous MSC-EVs studies. Further work will be useful to identify the EVs components underlying these activities and to determine their dose-response relationships and relevance in more complex skin models.

## Data Availability

The original contributions presented in the study are included in the article/Supplementary Material, further inquiries can be directed to the corresponding authors.
