# Supplementary material for: Integrated evaluation of umbilical cord mesenchymal stromal cell-derived extracellular vesicles across skin-related cellular and zebrafish models
Source: Front Bioeng Biotechnol. 2026 Sep 24;14:1939569. doi: 10.3389/fbioe.2026.1939569 (PMC13646170; doi:10.3389/fbioe.2026.1939569)
Supplement: Supplementary file 1 [file Supplementaryfile1.zip › Supplementary Table S1.docx]

**Table S1. qRT-PCR primer sequences.**

| **Gene** | **Primer sequence** |
| --- | --- |
| COL1A1 | Forward:5'-CTGGATGGATTGAAGGGACA-3'  Reverse:5'-CAACACGTCCTCTCTCACC-3' |
| COL3A1 | Forward:5'-AGGTCCTGCGGGTAACACT-3'  Reverse:5'-ACTTTCACCCTTGACACCCTG-3' |
| elastin | Forward:5'-GGGTTGTGTCACCAGAAGCA-3'  Reverse:5'-CAACCCCGTAAGTAGGAATGC-3' |
| HAS2 | Forward:5'-GTGGATTATGTACAGGTTTGTGA-3'  Reverse:5'-TCCAACCATGGGATCTTCTT-3' |
| MMP1 | Forward:5'-AAAATTACACGCCAGATTTGCC-3'  Reverse:5'-GGTGTGACATTACTCCAGAGTTG-3' |
| MMP3 | Forward:5'-CTGGACTCCGACACTCTGGA-3'  Reverse:5'-CAGGAAAGGTTCTGAAGTGACC-3' |
| MMP9 | Forward:5'-GGGACGCAGACATCGTCATC-3'  Reverse:5'-TCGTCATCGTCGAAATGGGC-3' |
| HO-1 | Forward:5'-AAGACTGCGTTCCTGCTCAAC-3'  Reverse:5'-AAAGCCCTACAGCAACTGTCG-3' |
| NQO1 | Forward:5'-GAAGAGCACTGATCGTACTGGC-3'  Reverse:5'-GGATACTGAAAGTTCGCAGGG-3' |
| SOD2 | Forward:5'-TTCAATAAGGAACGGGGACA-3'  Reverse:5'-ACACATCAATCCCCAGCAGT-3' |
| CAT | Forward:5'-TCCGGGATCTTTTTAACGCCATTG-3'  Reverse:5'-TCGAGCACGGTAGGGACAGTTCAC-3' |
| AQP3 | Forward:5'-GGAAGAGAATGTGAAGCTGGC-3'  Reverse:5'-GAAAGGGTGGATCGTGAAGG-3' |
| FLG | Forward:5'-GCTGAAGGAACTTCTGGAAAAGG-3'  Reverse:5'-GTTGTGGTCTATATCCAAGTGATC-3' |
| SRD5α1 | Forward:5'-CCTAAGGAATCTCAGAAAACCAGG-3'  Reverse:5'-GCATAGCCACACCACTCCATGA-3' |
| SREBP-1c | Forward:5'-GCCATGGATTGCACTTT-3'  Reverse:5'-CAAGAGAGGAGCTCAATG-3' |
| MITF | Forward:5'-TACAGTCACTACCAGGTGCAG-3'  Reverse:5'-CCATCAAGCCCAAAATTTCTT-3' |
| TYR | Forward:5'-GGCCAGCTTTCAGGCAGAGGT-3'  Reverse:5'-TGGTGCTTCATGGGCAAAATC-3' |
| TRP-1 | Forward:5'-GCTGCAGGAGCCTTCTTTCTC-3'  Reverse:5'-AAGACGCTGCACTGCTGGTCT-3' |
| TRP-2 | Forward:5'-AGACGGAACACTGGACTCTCAAG-3'  Reverse:5'-CAAGTTGCTCTGCGGTTAGGAAG-3' |
| VEGF | Forward:5'-GCACTGGACCCTGGCTTTAC-3'  Reverse:5'-GTTACAGCAGCCTGCACAGC-3' |
| IGF-1 | Forward:5'-TCGACATCCGCAACGACTATC-3'  Reverse:5'-CCAGGGCGTAGTTGTAGAAGAG-3' |
| FGF-7 | Forward:5'-CATGAACACCCGGAGCACTAC-3'  Reverse:5'-CACTGTGTTCGACAGAAGAGTCTTC-3' |
| GAPDH (human) | Forward:5'-AAAGCCTGCCGGTGACTAAC-3'  Reverse:5'-TTCCCGTTCTCAGCCTTGAC-3' |
| Gapdh (mouse) | Forward:5'-CCCTTAAGAGGGATGCTGCC-3'  Reverse:5'-TACGGCCAAATCCGTTCACA-3' |
