# Supplementary material for: Integrated evaluation of umbilical cord mesenchymal stromal cell-derived extracellular vesicles across skin-related cellular and zebrafish models
Source: Front Bioeng Biotechnol. 2026 Sep 24;14:1939569. doi: 10.3389/fbioe.2026.1939569 (PMC13646170; doi:10.3389/fbioe.2026.1939569)
Supplement: Supplementary file 1 [file Supplementaryfile1.zip › Supplementary Figure S.pdf]

Figure S1

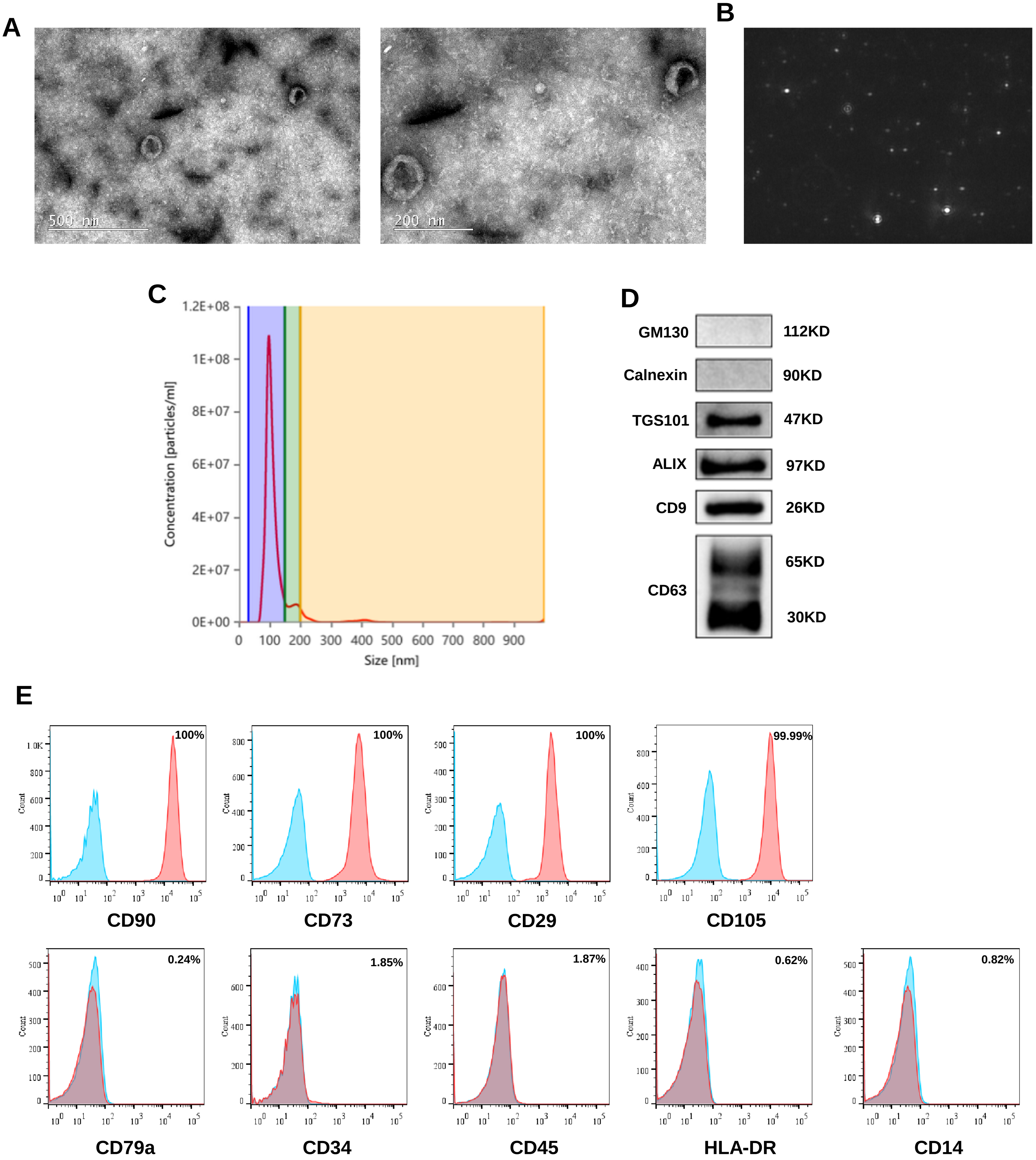
A
500 nm
200 nm
B
C
Concentration [particles/ml]
1.2E+08
1E+08
8E+07
6E+07
4E+07
2E+07
0E+00
0 100 200 300 400 500 600 700 800 900
Size [nm]
D
GM130 112KD
Calnexin 90KD
TGS101 47KD
ALIX 97KD
CD9 26KD
CD63 65KD 30KD
E
100%
CD90
100%
CD73
100%
CD29
99.99%
CD105
0.24%
CD79a
1.85%
CD34
1.87%
CD45
0.62%
HLA-DR
0.82%
CD14
Count

Figure S2

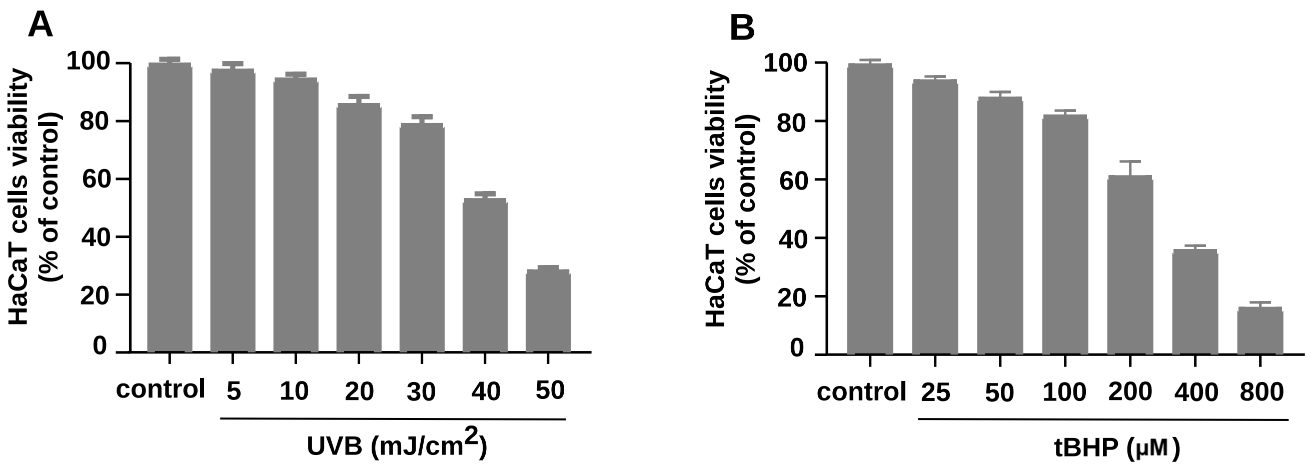
A
HaCaT cells viability (% of control)
100
80
60
40
20
0
control
5
10
20
30
40
50
UVB (mJ/cm2)
B
HaCaT cells viability (% of control)
100
80
60
40
20
0
control
25
50
100
200
400
800
tBHP (μM)

Figure S3

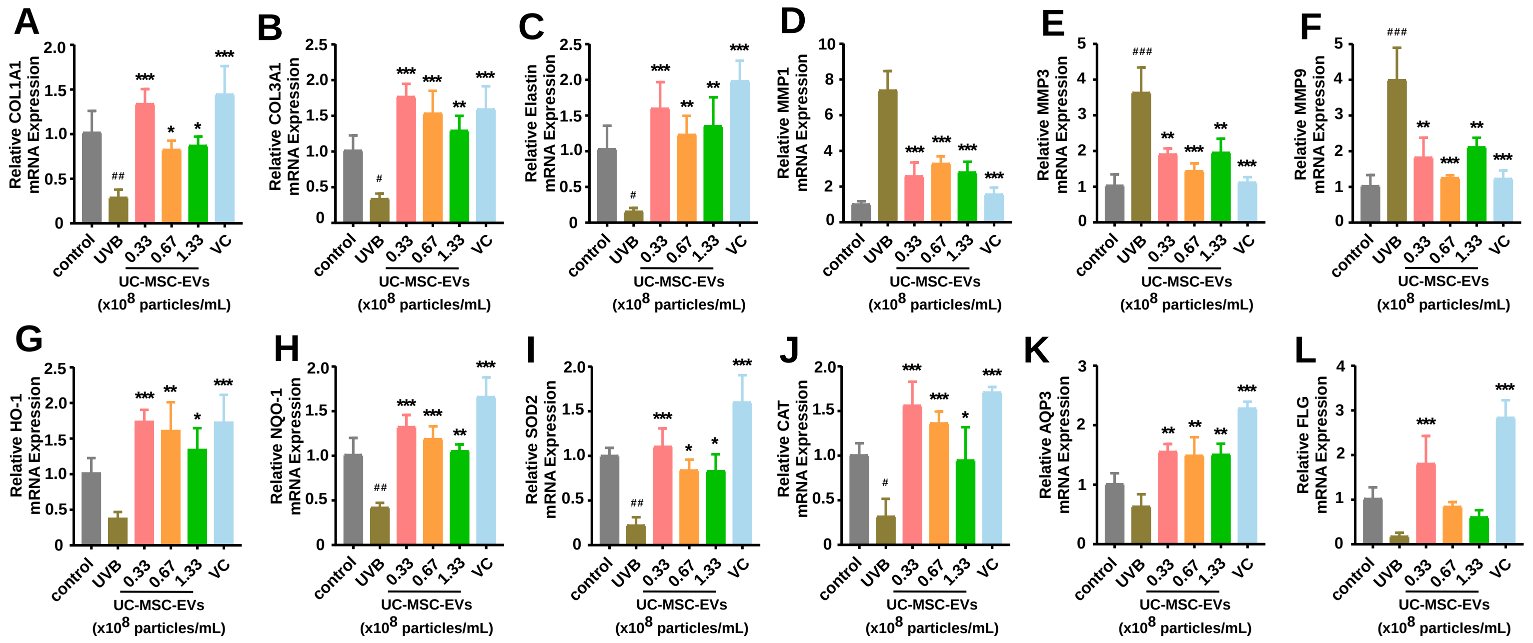

A
Relative COL1A1 mRNA Expression
B
Relative COL3A1 mRNA Expression
C
Relative Elastin mRNA Expression
D
Relative MMP1 mRNA Expression
E
Relative MMP3 mRNA Expression
F
Relative MMP9 mRNA Expression
G
Relative HO-1 mRNA Expression
H
Relative NQO-1 mRNA Expression
I
Relative SOD2 mRNA Expression
J
Relative CAT mRNA Expression
K
Relative AQP3 mRNA Expression
L
Relative FLG mRNA Expression
control
UVB
0.33
0.67
1.33
VC
UC-MSC-EVs
(x10^8 particles/mL)

Figure S4

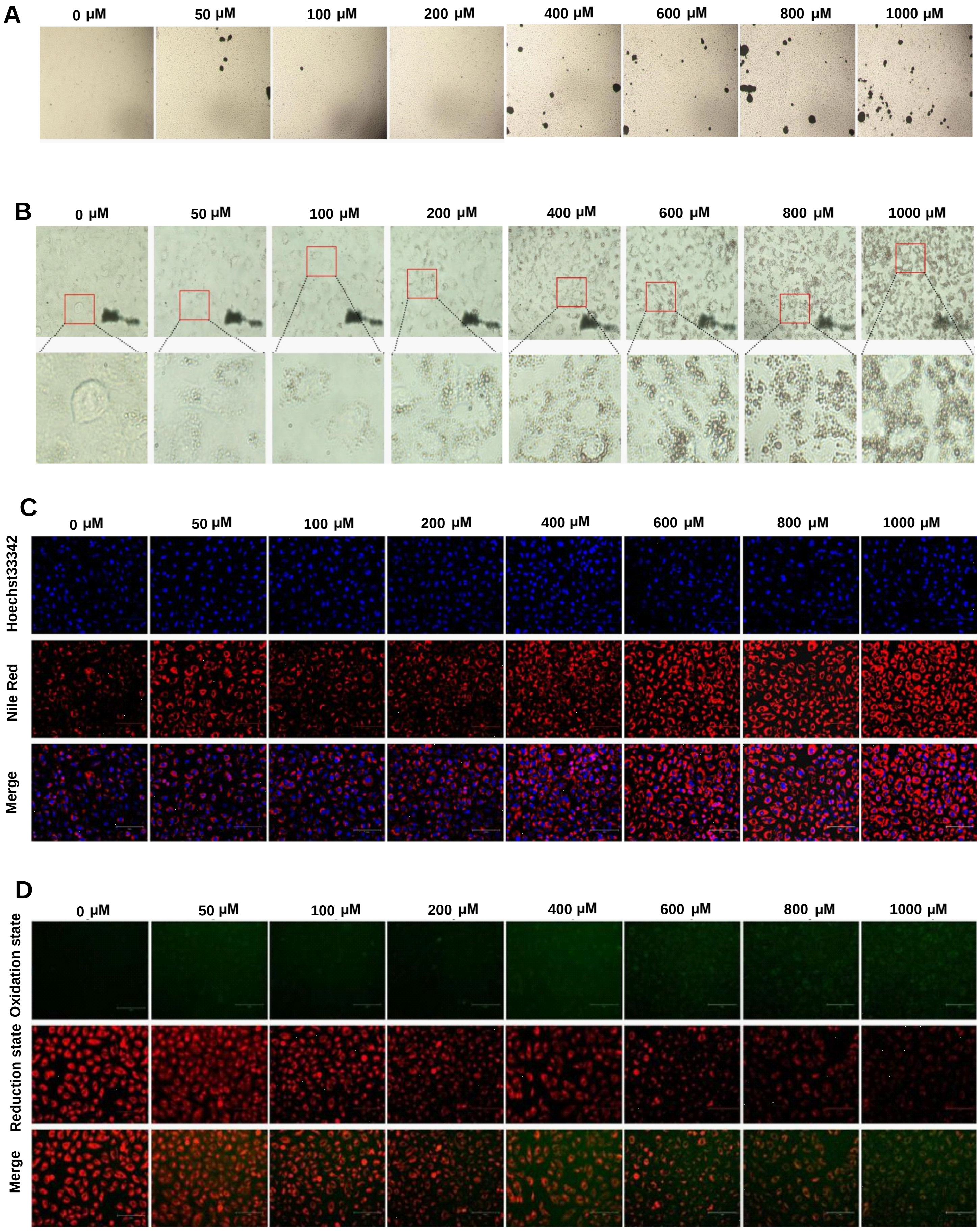
A
0 μM
50 μM
100 μM
200 μM
400 μM
600 μM
800 μM
1000 μM
B
0 μM
50 μM
100 μM
200 μM
400 μM
600 μM
800 μM
1000 μM
C
0 μM
50 μM
100 μM
200 μM
400 μM
600 μM
800 μM
1000 μM
Hoechst33342
Nile Red
Merge
D
0 μM
50 μM
100 μM
200 μM
400 μM
600 μM
800 μM
1000 μM
Oxidation state
Reduction state
Merge
